# Supplementary material for: Enhancing Anti-PD-1 Immunotherapy by Targeting MDSCs via Hepatic Arterial Infusion in Breast Cancer Liver Metastases
Source: Cancers (Basel). 2024 Nov 3;16(21):3711. doi: 10.3390/cancers16213711 (PMC11545300; doi:10.3390/cancers16213711)

Kim et al.
Supplementary Figure S1

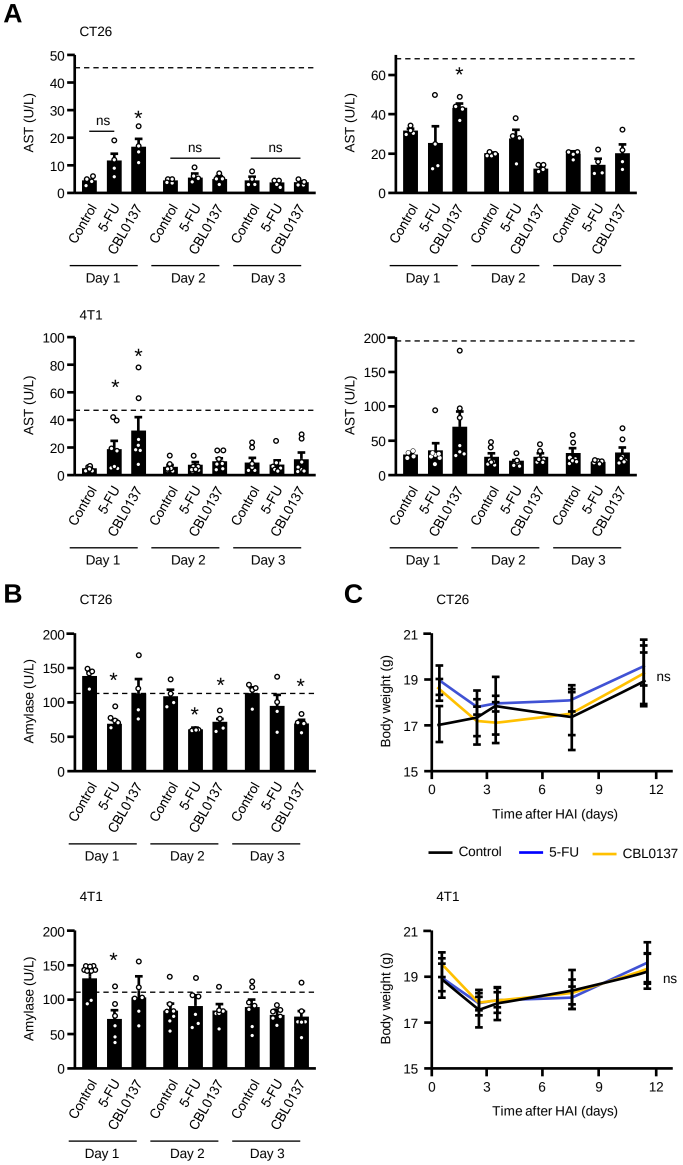

Supplementary Figure S1. Toxicity evaluation after HAI in CT26 and 4T1 liver tumor bearing mice. HAI with CBL0137 had tolerable and transient toxicity with no significant morbidity as noted by (**A**) liver enzymes, (**B**) amylase levels, or (**C**) changes in body weight. Dotted lines; normal values. Representative data between 2 experiments. CT26; *n = 4*, 4T1; *n* = 6, *; p< 0.05, ns; not significant by Student's *t* test, error bar; standard error of the mean. Body weight changes were recorded every 3 days after surgery with mice bearing CT26 (*n = 5*) and 4T1 (*n = 5*) tumor. Representative data between 2 experiments. ns; not significant by Student's *t* test, error bar; standard error of the mean.

Kim et al.

Supplementary Figure S2

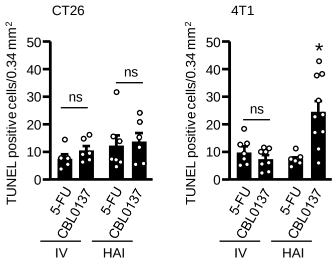

Supplementary Figure S2. The impact of HAI compared to intravenous (IV) injection targeting liver tumors. No statistically significant induction of TUNEL cells by either CBL0137 HAI or IV was found 5 days after each treatment in the setting of CT26 liver metastasis. HAI with CBL0137 was necessary for tumor response as measured by TUNEL staining (not noted by IV administration) in 4T1 liver metastatic tumors. pooled data from 2 independent experiments, ns; not significant, *; p< 0.05, $n \geq 6$, by Student's t test, error bar; standard error of the mean.

Kim et al.

Supplementary Figure S3

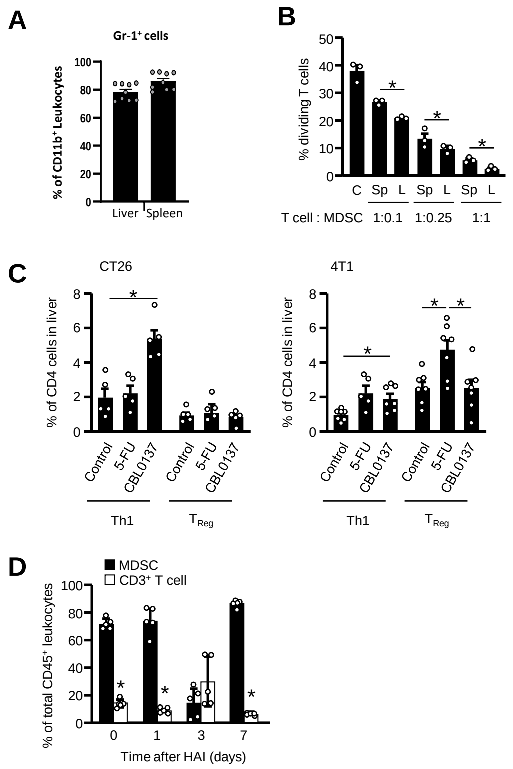

Supplementary Figure S3. The influence of CBL0137 HAI on immune cell subsets. (**A**) The population of Gr-1$^+$ cells after isolation from the liver and spleen with CD11b magnetic beads in bearing mice 4T1 liver metastases. Pooled data from 3 independent experiments, *n* = 9. (**B**) CD11b magnetic bead isolated MDSC and CD3/28 beads activated T cells were cocultured for suppression assays with different ratios. CD11b$^+$ cells were isolated from the spleen (Sp) and liver (L) and T cells were collected from the spleen. Representative data from 3 independent experiments, *n* = 3, *; $p < 0.05$ by Student's t test. (**C**) CD4 Th1 subsets are increased by CBL0137 without concomitant Treg population increases in the liver 5 days after HAI in mice bearing CT26 and 4T1 liver metastases; Pooled data from 3 independent experiments, *; $p < 0.05$, 4T1; *n = 7*, CT26; *n ≥ 5*; by Student's *t* test, error bar; standard error of the mean. (**D**) The reduction in MDSC noted from CBL0137 HAI is transient, peaking at day 3 after HAI and returning to baseline levels by day 7. 1 independent experiment, *; $p < 0.05$, *n = 5*, by Student's t test.

Kim et al.

Supplementary Figure S4

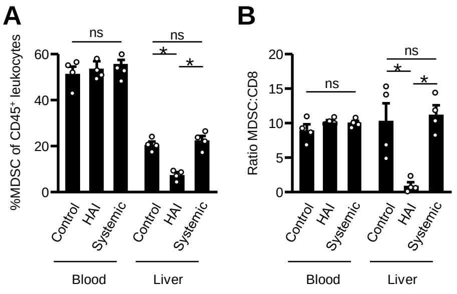

Supplementary Figure S4. A HAI is superior to a systemic intravenous injection for eradication of MDSC in the liver. (**A**) The composition of MDSC ($CD11b^+Gr\text{-}1^+$) population and (**B**) the ratios of MDSC:$CD8^+$ T cell in the blood and liver 5 days after HAI or systemic intravenous injection with 0.5 mg of CBL0137 in mice bearing 4T1 liver metastases, $n = 4$, ns; not significant, *; $p < 0.05$ by Student's t test, error bar; standard error of the mean. Representative data of 2 experiments.

Kim et al.

Supplementary Figure S5

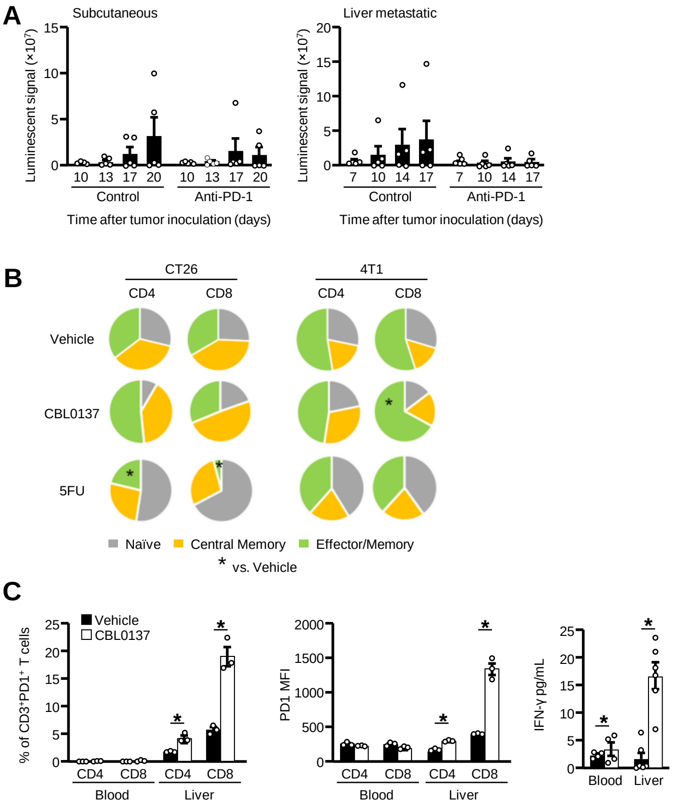

Supplementary Figure S5. CBL0137 HAI increases immune modulation effects with 4T1 liver metastases. (**A**) Mice bearing CT26 subcutaneous or liver metastatic tumor were treated with either vehicle or anti-PD-1 Ab as a single agent 3 times a week, and then luminescent signals were measured. Anti-PD-1 antibody treatment trended to decrease tumor growth in CT26 subcutaneous and liver metastatic tumors, but were not statistically significant. Pooled data from 2 different experiments, ns; not significant, *n = 5*, by Student's t test, error bar; standard error of the mean. (**B**) T cell activated status were analyzed as naïve ($CD62L^{+}CD44^{-}$), central memory (CM; $CD62L^{+}CD44^{+}$), and effector memory (EM; $CD62L^{-}CD44^{+}$) cell populations 5 days after each treatment in mice bearing CT26 and 4T1 liver metastases. Pooled data from 2 different experiments, *; p< 0.05, ns; not significant, *n ≥ 4*, by Student's t test, error bar; standard error of the mean. (**C**) Blood- and liver-derived leukocytes were analyzed by flow cytometry 5 days after HAI with either vehicle or CBL0137 in mice bearing 4T1 liver metastases. Representative data between 2 independent experiments, *; p< 0.05, *n = 3*, by Student's t test, error bar; standard error of the mean. IFN-γ concentrations 5 days after CBL0137 HAI in the blood and liver by ELISA. Pooled data from 2 different experiments, *; p< 0.05, *n ≥ 4*, by Student's t test, error bar; standard error of the mean.

Kim et al.

Supplementary Figure S6

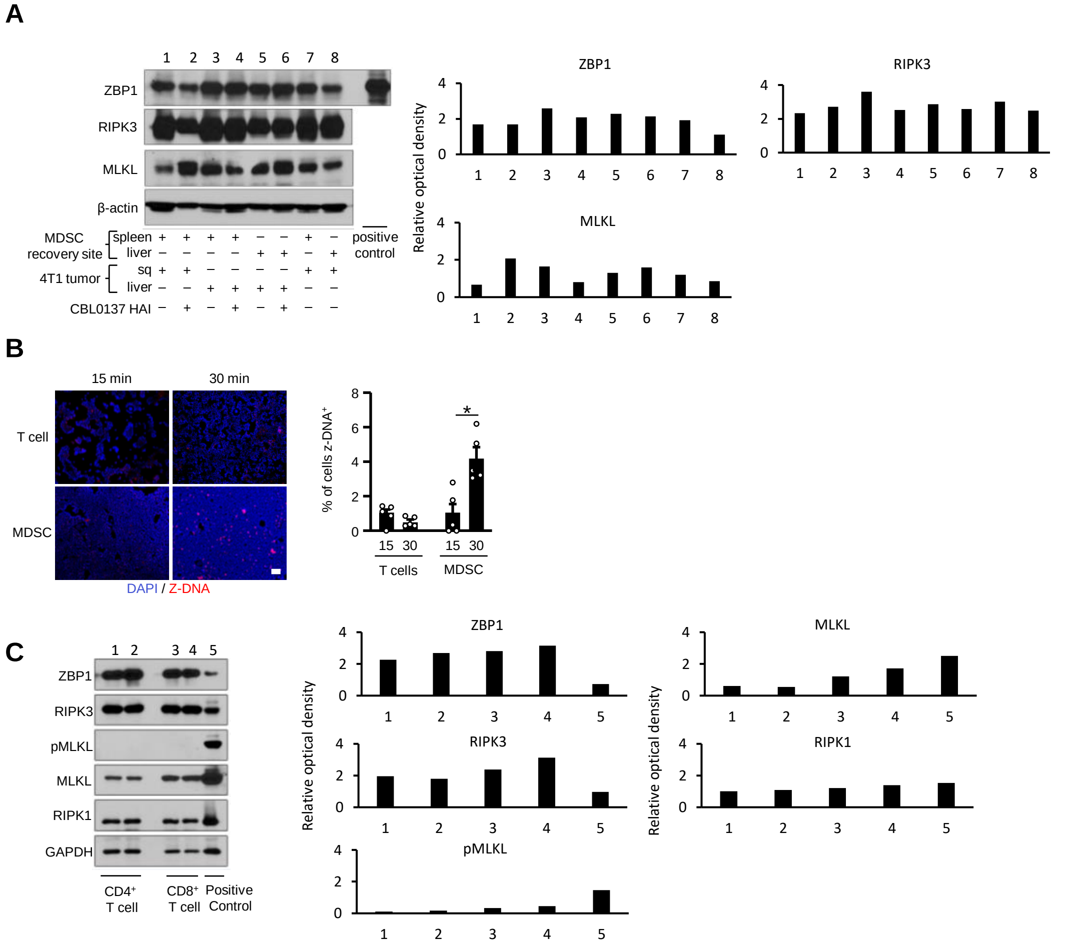

Supplementary Figure S6. Western blot and IF images for necroptosis machinery in MDSCs and T cells. (**A**) Western blot for the Z-DNA sensing protein ZBP1 and downstream molecules (RIPK3, MLKL, RIPK1) in T cells sorted into CD4 and CD8 populations shows consistently high levels of ZBP1, RIPK3, MLKL, and RIPK1 in all T cells recovered. (**B**) IF staining for Z-DNA after *ex-vivo* treatment with CBL0137 in T cell and MDSC. Representative data between 2 independent experiments, *; $p < 0.05$, $n = 5$, by Student's t test, error bar; standard error of the mean. (**C**) Western blot for the Z-DNA sensing protein ZBP1 and downstream molecules (RIPK3, MLKL, RIPK1) in MDSC.

Western Figure 3B

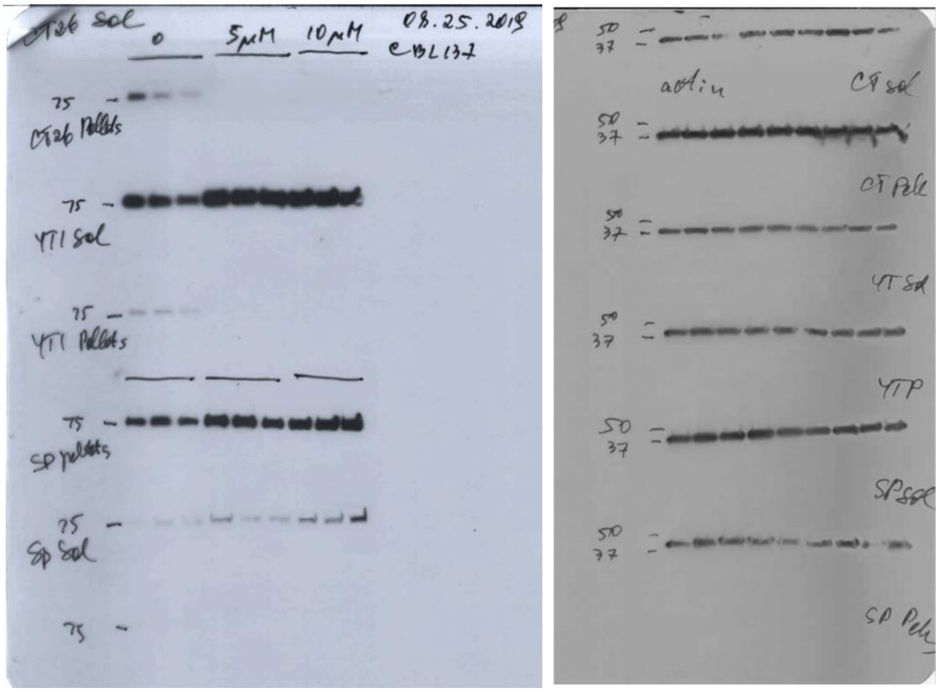

Western Figure 4A

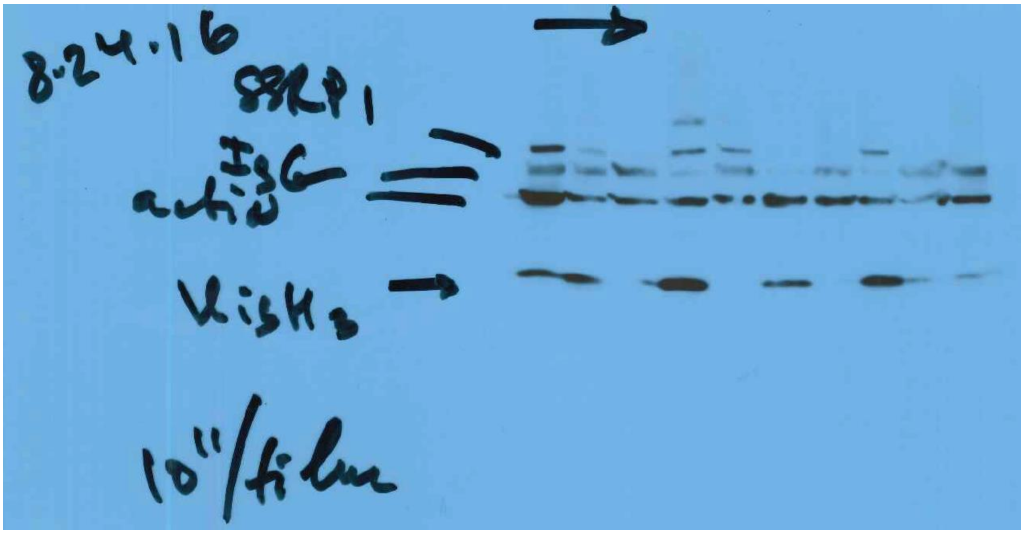

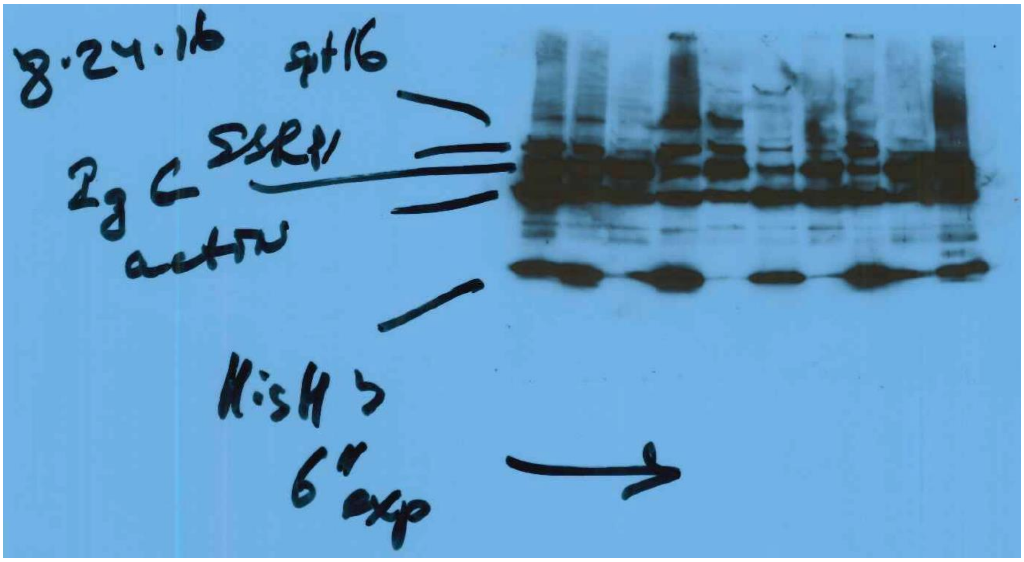

Western Figure 8E and Supplemental Figure 6A

β-actin

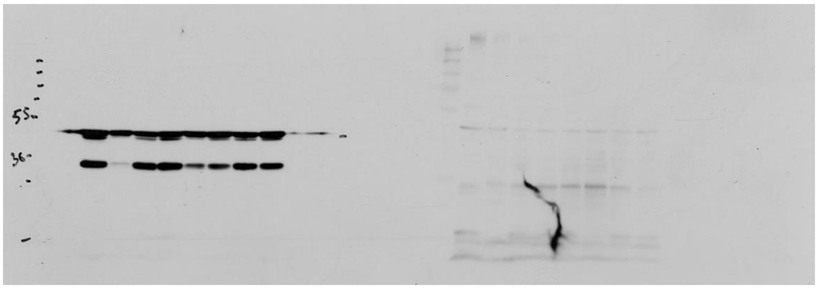

MLKL

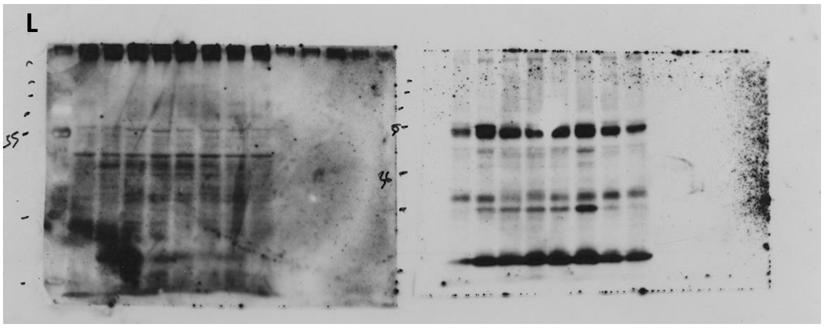

ZBP1

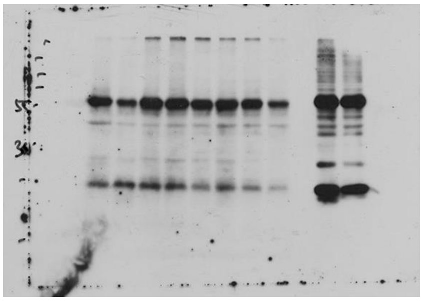

RIPK3

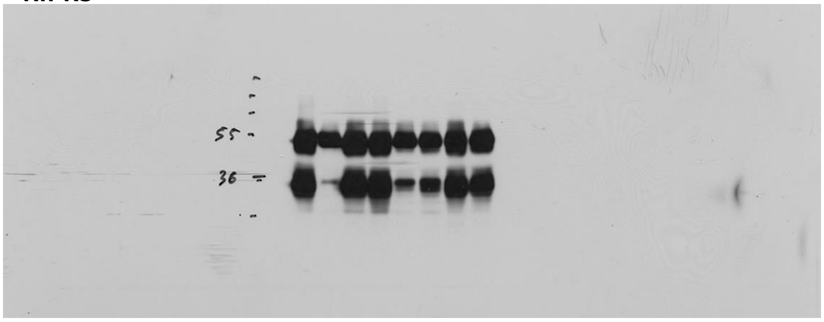

Western Supplemental Figure 6C

MLK
L

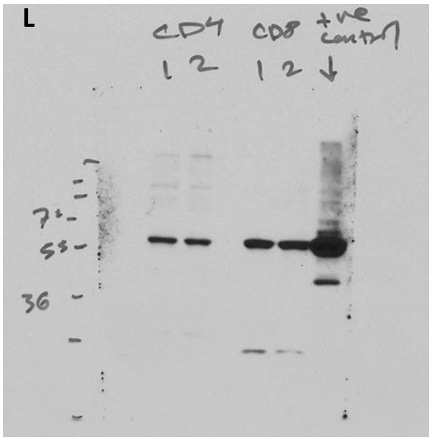

pMLKL

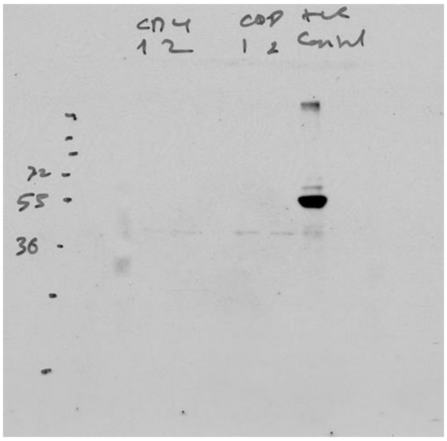

RIPK3

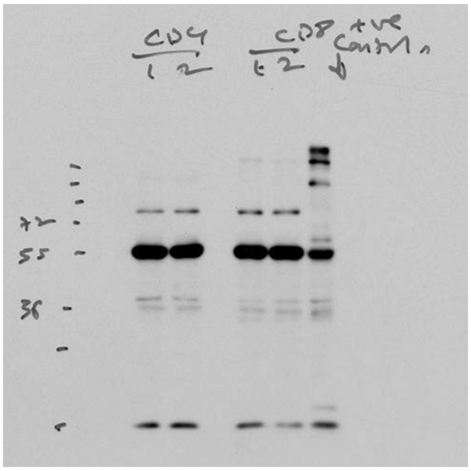

RIPK1, ZBP1, GAPDH

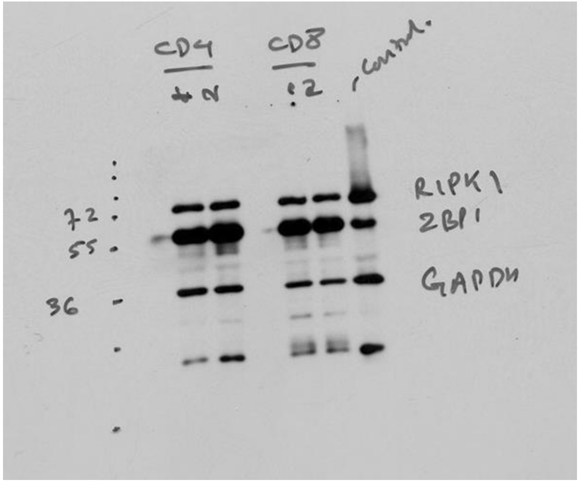

Supplement: Supplementary file 1 [file cancers-16-03711-s001.zip › cancers-3238853-supplementary.pdf]
